# Progress in Medicine

**Published:** 1897-09-04

**Authors:** 


### Progress in Medicine.

### DISEASES OF THE PHARYNX AND LARYNX.

(*Continued from page* 376.)

**New Growths—Benign.**—Massei,[17] of Naples, publishes an analysis of 500 cases of laryngeal neoplasms in his own practice. According to their nature and frequency, the 500 cases were:—

| | |
|---|---|
| Papillomata ... ... ... ... | 183 |
| Fibromata ... ... ... ... | 156 |
| Epitheliomata ... ... ... ... | 67 |
| Cystic tumours ... ... ... ... | 27 |
| Sarcomata ... ... ... ... | 10 |
| Myxomata ... ... ... ... | 6 |
| Encephaloid ... ... ... ... | 5 |
| Amyloid tumours ... ... ... | 4 |
| Adenomata ... ... ... ... | 2 |
| Gummata ... ... ... ... | 2 |
| Nature not determined in ... ... | 38 |
| | 500 |

Speaking of papillomas, Massei reminds us of the tendency to recurrence after removal. "But, happily, at the end of one or two years this progressive stage seems to stop, and endeavours to remove the growth are then successful." He has seen, however, a patient in whom recurrence took place twice, the first time after six years, and again another year later. On the other hand, he draws attention to the retrogressive phase, which can end in a complete disappearance of the tendencies, not rarely spontaneously, or observed in children. Often he prefers curettes to common forceps, when the growth is more infiltrated than prominent, and from local application of ichthyol (one to ten of distilled water) he has had, as concerns recurrence, better results than from caustics. But if a laryngoscopic examination is not possible, and it is necessary to correct the difficulty of breathing, he is convinced (and insists now more than ever) that simple tracheotomy may be sufficient.

If after a time (one or two years) the tumour persists, there is, then, no contra-indication for thyrotomy; but the practice of beginning by this (as surgeons, and not specialists do) is erroneous according to Massei, and he mentions a case in point, a girl 11½ years old, who was last September operated on (thyrotomy) on account of a severe dyspnœa for laryngeal papillomata. Breath restored; voice only improved after operation, and a month after again aphonia. For this the little patient applied to him in December, and he could easily see, on the edge of the left vocal cord and in the sub-glottic space, papillary growths, which he removed by laryngoscopic way, with complete return of the voice.

The second place, after papillomata, is taken by fibromata. He explains the reason of this frequency, it being not a question of true fibromata, but of a variety of "cystic fibromata." Pathologically they are benign, though the rapid augmentation of the interstitial spaces, or a hæmorrhage may give rise to alarming symptoms on account of a quick enlargement of the growth. In general such neoplasms, which take a bi-lobulated or tri-lobulated appearance, of a red contour, often very strong, with a well-marked pedicle, do not reach a considerable volume; very seldom do they recur when extirpated. As regards cystic tumours, the prevalent seat is the vocal cords or the epiglottis; they resemble mucous polypi in aspect. Their origin from the vocal cords confirms the idea that they may be considered as true mucous cystic tumours arising in consequence of retention of the secretion of mucous glands by obliteration of their ducts. But the contents may be serum, blood, fibrin, or transformed secretive products. They readily recur after removal hence the necessity of cauterising the base. Myxomas are small, and do not tend to recur.

Amyloid tumours are simple curiosities. In the 500 cases they appear only four times. Amyloid tumours may be multiple, small in size, round in form, or large; but they may be seen also isolated on the vocal cords. Twice it was a microscopic examination which instructed Massei. In his first two cases the windpipe was also the seat of little growths, and severe dyspnœa was present. Once tracheotomy was performed; in the second no operation was done, and the patient died. In the third and fourth instances the growths were small, round, similar to cystic fibromata, placed over the vocal cords. Extirpated, they did not recur. Dr. G. Martuscelli, who undertook a microscopical examination, informed him that they consisted, for the most part, of a matter which coloured well under the special iodine reaction. Massei concludes that amyloid tumours may be separated into two classes—those in which, according to the opinion expressed by Ziegler, the presence of amyloid substance occurs in form of multiple growths, not only in the larynx, but also at the base of the tongue and elsewhere, and may be attributed to circulatory troubles, and those in which the accidental presence of amyloid substance may have a more local significance. But it is also highly probable, says Massei, that if careful investigations were made in all cases of laryngeal growths, the presence of amyloid substance would be detected oftener. Adenomata may reach a considerable size; they have been seen over the vocal cords, the epiglottis, the exterior surface of arytenoid cartilages. They have no characteristic appearance, but may resemble a tonsil and recur (as in one of his two cases).

The treatment of papillomas by phenol ricinate has been investigated by Th. Heryng.[18] He remarks that, however thoroughly and radically performed, papillomas frequently recur. The cautery, instead of preventing, seems rather to promote recurrence. Caustics, such as chromic acid, nitrate of silver, mineral acids, zinc chloride, and salicylic acid, have all disadvantages; even lactic acid is no exception to this rule. Disappointed with these, Heryng tried sulphoricinate of phenol, and found that it had the effect not only of preventing recurrence after operation to an extraordinary degree, but also in certain cases of removing the growths without previous operation. Five cases are cited in illustration of these facts; of these we give one. M. C., aged 40, had been operated on twelve times by various doctors during two years. First seen by Heryng on October 1st, 1894. Papillomas on both vocal cords, left processus vocalis, and both aryepiglottic folds. In several sittings the growths on the vocal cords and vocal process were removed with sharp forceps, and 30 per cent. of the phenol rubbed in. The growths on the aryepiglottic folds, simply rubbed with the same solution, disappeared after four applications. Phenol treatment carried on for six weeks. Voice quite clear; remained so till January 7th, 1896. Very small growth found at anterior commissure; rubbed three times, disappeared; no recurrence. He finds that growths on the aryepiglottic folds and cartilage of Santorini disappear under simple painting; elsewhere they must be first operated on. This is due to the nature of the epithelial covering.

Bond[19] describes a case in a female patient, aged 30, who had complained of sore throat for the preceding nine months, and of marked and increasing difficulty in swallowing. She looked very ill, and was constantly retching and trying to swallow the abundant mucus present. A large, sloughing, cauliflower-like mass was seen to be occupying the lower pharynx, behind the epiglottis, and was traced by palpation to the upper end of the œsophagus. It was removed by snaring, and on microscopic examination by Hewett it proved to be a fibroma.

The question of preliminary tracheotomy in operations on the upper air passages is discussed by F. W. Murray,[20] of New York. Shall we use the tampon cannula, or shall we insert an ordinary tube and tampon the pharynx? The answer given is that it depends partly on individual preference and partly on the location of the disease. In operations involving the upper jaw, tongue, tonsils, or upper pharynx respectively, Murray prefers the ordinary tracheotomy tube, combined with tamponnade of the pharynx. It allows of a smaller wound of the trachea, and avoids the possibility, however remote, of blood and septic secretions lodging in the larynx above the tampon and causing septic pneumonia. In total extirpation of the larynx or of the lower pharynx, together with the larynx, the tampon cannula is indicated, and of these we have a choice between Trendelenburg's, Hahns', Kocher's, and Gerster's. The sponge cannulas soon become septic, and should therefore be replaced immediately after operation by an ordinary tracheotomy tube.

**Singer's Nodules.**—Kanthack[21] recently demonstrated a section of a Singer's nodule, hardened and fixed in formalin. The specimen showed marked interstitial myositis, and disproved the glandular origin of these nodules. Semon stated, in reference to this case, that he had no doubt that the affection was of an inflammatory character, but that Dr. Kanthack's contribution taught something quite new, viz., that the inflammation actually extended to the musculus vocalis.

**Malignant Growths.**—A case well illustrating the importance of duly appreciating the earliest manifestations of malignant disease of the larynx, has been recorded by Middlemass Hunt.[22] The patient, a man of 56, had been slightly hoarse for about a year. Laryngoscopic examination showed marked congestion of the left vocal cord, and on its edge, in the anterior third, was a small white, flat papilloma, which seemed to invade rather than grow from the surface of the cord. There was perfect movement of both cords in phonation and inspiration. The growth was diagnosed as malignant from—(1) its white colour, (2) its invasion of the substance of the cord, (3) the soft ulcerated appearance of its surface, (4) the congestion of the cord on which it was situated, and (5) the age of the patient. Thyrotomy was performed after a course of iodide of potassium, and the left cord excised. The growth, which was no larger than a millet seed, presented the microscopic character of an early epithelioma. It had not recurred six months later, the patient being well and having an excellent voice.

**Trachea.**—Massei[23] draws attention to a certain small number of cases, in which the introduction of the intubation tube or the performance of tracheotomy has been followed by the escape of a considerable amount of pus. The cases have been characterised clinically by an acute stenosis of the larynx in children between two and seven, accompanied by symptoms and laryngoscopic evidence of paralysis of one vocal cord. The author is inclined to regard these cases as analogous to idiopathic post-pharyngeal abscess; and, in the absence of post mortem evidence, this opinion is based on the hypothesis that the condition is due to a purulent adenitis affecting the superior group of the peritracheal chain of the lymphatic glands.

**Roentgengraphy.**—An extremely interesting case of a foreign body removed from the œsophagus by œsophageal forceps, guided by the aid of the fluoroscope, is recorded by H. Beeckman Delatour,[24] of Brooklyn.

Scheier[25] has made experiments with the fluorescent screen and with the photographic plate, and finds that a bead in the nose could be readily made out, but that fruit stones were almost invisible. Examination of the sinuses revealed the fact that, by means of a photographic proof, the outlines of each of the sinuses were clearly defined. Foreign bodies have been discovered in the tonsils, and the method of procedure is to incline the head on the shoulder opposite to that on which the tonsil is situated; the organ is then placed in such a position that nothing intercepts the kathodal rays directed upon it. Tuberculous glands, surrounding the larynx and trachea, were plainly visible.

[17] Tri. State Med. Journ., Feb., 1897. [18] Therap. Monat., March, 1897; Journ. of Laryng., May. [19] Proc. Laryng. Soc. of London, 1897. [20] Annals of Surgery, May, 1897. [21] Proc. Laryng. Soc. of Lond., Jan., 1897. [22] Ibid. [23] Rev. Hebd. de Lar. Ot. et Rhin., Feb. 13, 1897; Journ. de Laryng., Mai., 1897. [24] Med. Record, May 1, 1897. [25] Arch. de Laryn., Nov. and Dec., 1896; Med. Chron., Feb., 1897.

